# Methodological Reporting of Randomized Trials in Five Leading Chinese Nursing Journals

**DOI:** 10.1371/journal.pone.0113002

**Published:** 2014-11-21

**Authors:** Chunhu Shi, Jinhui Tian, Dan Ren, Hongli Wei, Lihuan Zhang, Quan Wang, Kehu Yang

**Affiliations:** 1 Evidence-Based Medicine Center, Lanzhou University, Lanzhou, China; 2 Key Laboratory of Evidence Based Medicine and Knowledge Translation of Gansu Province, Lanzhou University, Lanzhou, China; 3 School of Basic Medical Sciences, Lanzhou University, Lanzhou, China; 4 The First Clinical Medicine School, Lanzhou University, Lanzhou, China; 5 The Second Clinical Medical School, Lanzhou University, Lanzhou, China; Cardiff University, United Kingdom

## Abstract

**Background:**

Randomized controlled trials (RCTs) are not always well reported, especially in terms of their methodological descriptions. This study aimed to investigate the adherence of methodological reporting complying with CONSORT and explore associated trial level variables in the Chinese nursing care field.

**Methods:**

In June 2012, we identified RCTs published in five leading Chinese nursing journals and included trials with details of randomized methods. The quality of methodological reporting was measured through the methods section of the CONSORT checklist and the overall CONSORT methodological items score was calculated and expressed as a percentage. Meanwhile, we hypothesized that some general and methodological characteristics were associated with reporting quality and conducted a regression with these data to explore the correlation. The descriptive and regression statistics were calculated via SPSS 13.0.

**Results:**

In total, 680 RCTs were included. The overall CONSORT methodological items score was 6.34±0.97 (Mean ± SD). No RCT reported descriptions and changes in “trial design,” changes in “outcomes” and “implementation,” or descriptions of the similarity of interventions for “blinding.” Poor reporting was found in detailing the “settings of participants” (13.1%), “type of randomization sequence generation” (1.8%), calculation methods of “sample size” (0.4%), explanation of any interim analyses and stopping guidelines for “sample size” (0.3%), “allocation concealment mechanism” (0.3%), additional analyses in “statistical methods” (2.1%), and targeted subjects and methods of “blinding” (5.9%). More than 50% of trials described randomization sequence generation, the eligibility criteria of “participants,” “interventions,” and definitions of the “outcomes” and “statistical methods.” The regression analysis found that publication year and ITT analysis were weakly associated with CONSORT score.

**Conclusions:**

The completeness of methodological reporting of RCTs in the Chinese nursing care field is poor, especially with regard to the reporting of trial design, changes in outcomes, sample size calculation, allocation concealment, blinding, and statistical methods.

## Introduction

The randomized controlled trial (RCT) is the most appropriate design for evaluating the effectiveness of a nursing intervention among eligible primary study designs for evidence-based practice [1]. Evidence-based nursing practitioners believe that randomization and unbiased allocation of interventions assure methodological quality and the subsequent findings of RCTs [2], [3]. However, well-conducted trials may be reported poorly [4], and failing to report all of the study's methods, details, and findings, especially for RCTs, seriously distorts the evidence base for clinical decision-making [5].

As a set of evidence-based reporting guidelines for RCTs, the Consolidated Standards of Reporting Trials (CONSORT) [6] was developed to improve the transparency of reporting and interpreting RCTs, minimize biased conclusions, ultimately facilitate clinical decision making, and indirectly drive better design of trials. The CONSORT guidelines have been associated with improving the quality of RCT reporting [7]. To date, CONSORT has been accepted by 600 medical journals, including 20 nursing care journals in English [8], and it has been revised twice, in 2001 and 2010 [9].

For RCTs in various disciplines (e.g., diagnostic procedures, interventions, cancer trials, and alternative medicine), the reporting quality has been assessed via CONSORT [5] to ensure their transparency, and found widespread inadequate reporting of methodological issues. However, evidence is still deficient on the reporting quality of RCTs in nursing care, especially in China. Existing reviews have focused on only a specific discipline [10], [11], were not conducted systematically [12], [13], used a modified CONSORT checklist according to the purposes of the study [14], or focused on only a specific methodological issue [15], [16]. This study was thus conducted to investigate (1) the adherence to methodological reporting complying with CONSORT on Chinese nursing RCTs, and (2) the correlation between CONSORT adherence and key indicators regarding characteristics and methodological factors.

## Methods

### Information Source

According to their total number of citations and impact factors (IFs) in the “Chinese S&T Journal Citation Reports 2012” [17], we selected the following five leading Chinese nursing journals(see Appendix S1): Chinese Journal of Nursing (CJN, 1954–), Chinese Journal of Modern Nursing (CJMN, 1995–), International Journal of Nursing (IJN, 1980–), Chinese Journal of Practical Nursing (CJPN, 1985–) and Journal of Nurses Training (JNT, 1986–). In June 2012, a comprehensive electronic search strategy for the Chinese Bio-Medicine disc (CBM; see Appendix S2) was performed using “randomized controlled trial” or “clinical trial” as keywords, limited to these five targeted journals.

### Eligibility Criteria

#### Inclusion criteria

1. Citations claiming to be RCTs in the titles and/or abstracts, according to the “Cochrane definitions and criteria for randomized controlled trials and controlled clinical trials” [18].
2. Controlled trials with two comparators.
3. Trials of both nursing interventions (e.g., drug interventions, social and psychological interventions, traditional Chinese medicine interventions, complex interventions) and non-nursing interventions (e.g., nursing education program interventions).

#### Exclusion criteria

1. “Quasi-random” trials: when “random” was mentioned with a high-risk randomization method, such as alternation, assignment based on date of birth, case record number, or date of presentation.
2. Irrelevant study reports: reports that are published in a summary form, reviews of randomized trials, and randomized trials on animals, even though they may be reported as “random.”
3. Other study designs, such as cohort study and case series report.

### Study Selection and Data Extraction

All citations from the CBM were allocated evenly to three units. By first screening titles and abstracts and then screening fulltexts, three groups consisting of two authors each selected eligible studies from these units of citations independently. When a disagreement arose in any of groups, another group would participate to arrive at an agreement. Then, two authors extracted relevant information and data from the included studies. A well-designed extraction form (Appendix S3) was formulated to assess the reporting quality of RCTs according to CONSORT [6]. The extraction form included (1) general characteristics [5] (i.e., journal name, IF, publication date, number of authors, multicenter trials, types of interventions, sample size, and funding source) and a description of the interventions [19], including length of descriptions of interventions and controls in words and the reproducibility of the interventions, which authors evaluated by assessing whether the intervention was described in enough detail to be reproducible; (2) CONSORT methodological items; and (3) methodological quality measurements [20], including allocation concealment and intention-to-treat (ITT) analysis. The overall CONSORT methodological items score was the total score of 17 items with response options of “yes” (scored as 1) or “no” (scored as 0) on whether the component had been reported as recommended. Items were scored as “no” (i.e., 0) if the item was only partly met by the research report. Disagreements during data extraction were resolved by discussion.

### Quality Control

We implemented rigorous quality control throughout the study. Before selecting the eligible studies, investigators were trained to distinguish RCTs from irrelevant study designs using the definitions from the Centre for Evidence-Based Medicine, Oxford, UK (University of Oxford) [21]. Before data extraction, the extraction form (Appendix S3) following CONSORT guidelines was established via three rounds of pre-survey and team discussion.

### Data Analysis

Using SPSS 13.0, we performed a descriptive analysis, obtaining frequencies and percentages for categorical variables, and means, medians, standard deviations, and inter-quartile ranges (IQR) for continuous variables. Then, exploratory regression was conducted to examine whether general and methodological characteristics are associated with reporting quality, as measured by the CONSORT methodological items score. Univariate regression was performed for each potentially associated variable, and variables with P values lower than 0.25 were then included in a stepwise backward linear regression. Any P value lower than 0.05 was regarded as statistically significant. In this study, the sample sizes of the trials were included to power the exploratory linear regression.

## Results

### Study Inclusion

**Figure 1:**
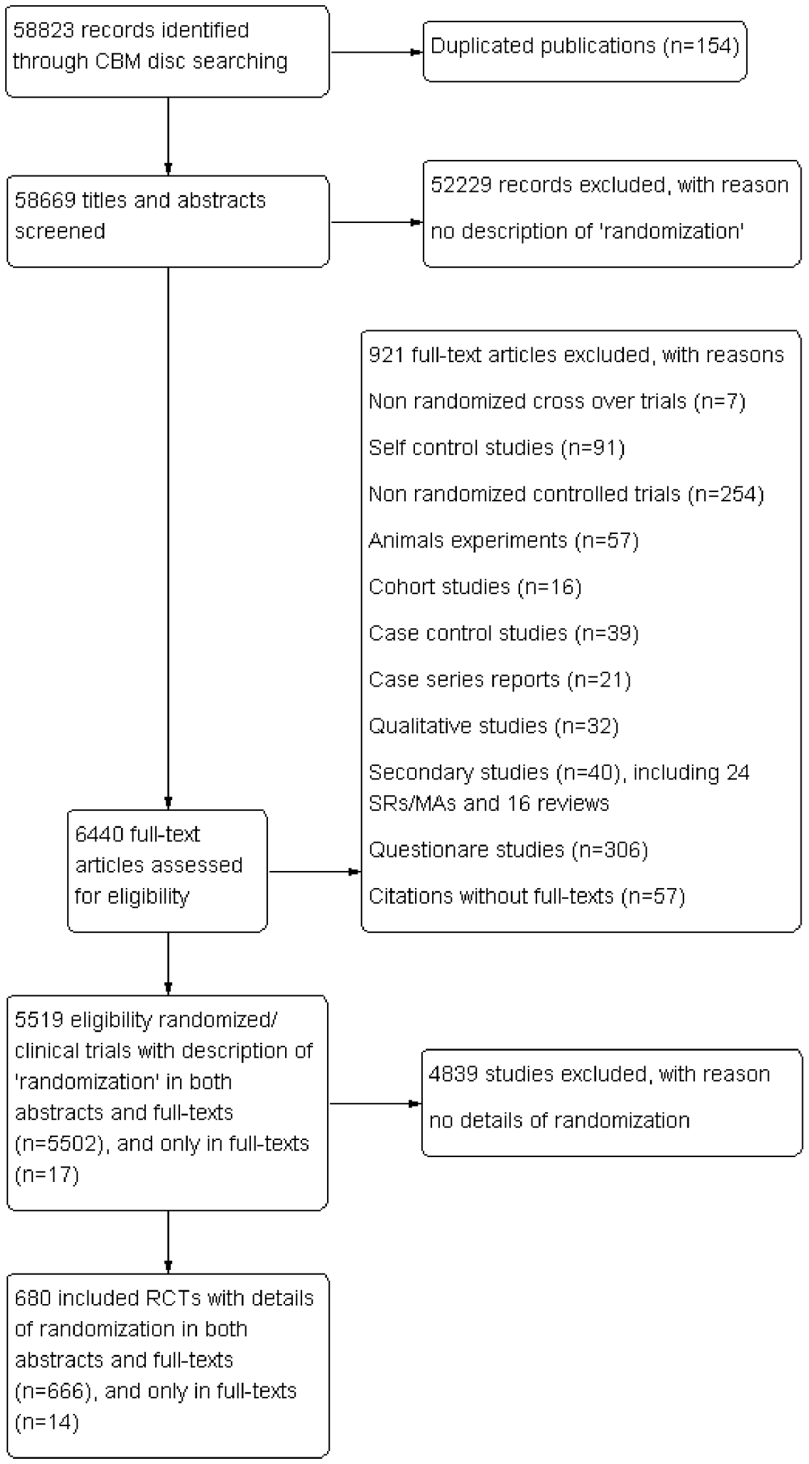
Flow diagram of included studies.

In total, 58,823 citations published in the five leading nursing journals from 1954 to June 2012 were retrieved via the “Journal Search Engine” function of the CBM disc. After screening abstracts and fulltexts, 5519 fulltexts that mentioned “random” were initially identified. Among them, 5502 trials described “randomization” in both abstracts and fulltexts, and 17 described “randomization” in only the fulltexts. Six hundred and eighty trials met the criteria for “details of randomization” and were included in the final analysis. Figure 1 depicts a flow diagram of the inclusion decisions.

### Characteristics of Included Studies

Most of the included trials (99.9%) were published after the 2001 CONSORT (Appendix S4). Furthermore, 27.6% (188/680) of trials were published in CJN and JNT, which had higher IFs than the average (mean ± SD  = 1.20±1.05); 61% (415/680) of trials were reported by no more than three authors (i.e., the median number of authors). Two point five percent (17/680) of trials reported a collaboration between multicenter studies. However, 21.6% of these trials did not specify the number of clinical trial centers. Most trials (83.4%) studied the implementation of non-pharmacological interventions. The median sample size of the included studies was 90 (IQR  = 62.75). Most trials (75.6%) did not mention financial support. By our judgment, 85.4% of trials provided enough details in the description of interventions to facilitate replication; however, only 48.2% of control group protocols were sufficiently described. Nisnety percent of trials from CJPN described the control group protocol as “the control group used standard nursing inventions for this disease.” There were significant differences in the number of words used to describe the interventions and controls (median  = 215.0 and IQR  = 422.75 for interventions, and median  = 32.0 and IQR  = 47.0 for controls, Z = −21.192, P<0.001). For methodological quality measurements, only two trials (0.3%) reported the details of the allocation concealment mechanism; 85.3% of trials had no dropouts, and 5.7% conducted per protocol analysis. Table 1 depicts the characteristics of the included studies.

**Table 1:** Characteristics of Included Studies.

Predictors		n* (Total N = 680)	Percent (%)
Journal	CJMN	255	37.5
	JNT	73	10.7
	CJN	115	16.9
	IJN	106	15.6
	CJPN	131	19.3
Publication year§	–1996	1	0.1
	1996–2000	0	0
	2001–2005	44	6.5
	2006–2010	373	54.9
	2010–	262	38.5
No. of authors		Median = 3	IQR = 3
	≤3 authors	415	61
	>3 authors	265	39
Multicenter trials	Yes	17	2.5
	No	516	75.9
	Unclear	147	21.6
Type of interventions	Non-pharmacological interventions	567	83.4
	Pharmacological interventions	113	16.6
Sample size		Median = 90	IQR = 62.75
Funding support	Industry support	3	0.4
	Public support	163	24
	Unclear	514	75.6
Length of descriptions of interventions in words		Median = 215.0	IQR = 422.75
Reproducibility of interventions	Yes	581	85.4
	No	99	14.6
Length of descriptions of controls in words¶		Median = 32.0	IQR = 47.0
Reproducibility of control	Yes	328	48.2
	No	352	51.8
Allocation concealment	Yes	2	0.3
	No/unclear	678	99.7
ITT analysis	Yes/no drop outs	580	85.3
	Per protocol analysis	39	5.7
	Unclear	61	9

*Items are reported as frequency and proportion for categorical variables and are otherwise specified for continuous variables.

§ CONSORT was published in 1996 and revised twice in 2001 and 2010, respectively; the number of RCTs in each publication year is depicted in Appendix S4.

¶ There is a significant difference between the number of words used to describe interventions and controls,as indicated by two related-samples tests (Z = −21.192, P<0.001);

IQR indicates interquartile range.

### Reporting of CONSORT Methodological Items

When screening abstracts rather than fulltexts, 97.9% of RCTs could be identified. Only two trials (0.3%) reported “randomized control trial” in the title. Overall, the mean CONSORT methodological score was 6.34±0.97 out of 17 sub-items. Additionally, in order to explore the influence of the score of “blinding (11a, 11b)”, an analysis excluding data on blinding showed that the mean and SD of the CONSORT score were 6.28 and 0.94 respectively, which were similar to the mean score obtained while including data on blinding. No RCT reported descriptions and changes in “trial design,” changes in “outcomes” and “implementation,” or descriptions of the similarity of interventions for “blinding.” For the description of trial design, 99.7% of the studies used a parallel design while 0.3% used a crossover design. Sub-standard reporting was also found regarding the details of “settings of participants” (13.1%), “type of randomization sequence generation” (1.8%), calculation methods of “sample size” (0.4%), explanation of any interim analyses and stopping guidelines of “sample size” (0.3%), additional analyses of “statistical methods” (2.1%), and targeted subjects and methods of “blinding” (5.9%). In addition, no central randomization that implied allocation concealment was reported. Only two studies conducted allocation concealment through sequentiallynumbered opaque sealed envelopes. More than 50% of trials described randomization sequence generation, the eligibility criteria of participants, “interventions,” and definitions of “outcomes” and “statistical methods.” Table 2 depicts the reporting of individual CONSORT methodological items.

**Table 2:** Reporting of individual CONSORT methodological items.

Methodological items		n (Total N = 680)	Percent (%)
RCT*		666	97.9
Title		2	0.3
Trial design	Description (3a)	0	0
	Parallel design¶	678	99.7
	Crossover design¶	2	0.3
	Changes and reasons (3b)	0	0
Participants	Eligibility criteria (Item 4a)	370	54.4
	Settings (4b)	89	13.1
Interventions	Item 5	643	94.6
Outcomes	Definition (6a)	484	71.2
	Changes(6b)	0	0
Sample size	Calculation (7a)	3	0.4
	Interim analyses (7b)	2	0.3
Randomization sequence generation	Methods of randomization (8a)		
	Coin toss	47	6.9
	Computerized random number generator	28	4.1
	Drawing lots	61	9
	Random number table	543	79.9
	Minimization	1	0.1
	Type of randomization (8b)		
	Blocked randomization	10	1.5
	Simple randomization	668	98.2
	Stratified randomization	2	0.3
Allocation concealment mechanism	Item 9	2	0.3
Implementation	Item 10	0	0
Blinding	Targeted subjects and methods (11a)		
	Double blinding	4	0.6
	Single blinding	36	5.3
	Unclear	640	94.1
	Similarity of interventions (11b)	0	0
Statistical methods	Statistical methods (12a)	627	92.2
	Additional analyses (12b)	14	2.1
Overall score**		Mean = 6.34	SD = 0.97

*RCTs according to abstracts.

¶ Parallel and crossover design are judged by authors.

**In order to explore the influence of the score of “blinding (11a, 11b),” we excluded data on blinding and found the mean and SD to be 6.28 and 0.94, respectively, which are similar to the mean and SD obtained when including data on blinding.

### Predictors Associated with CONSORT Methodological Score

We explored the association of predictors, including general characteristics and methodological quality measurements, with CONSORT methodological score with a linear regression and found that publication year and ITT analysis were weakly associated with CONSORT score. Each predictor was associated with a small increase of 0.047 and 0.601 points to the overall CONSORT methodological score. The two predictors associated with CONSORT score (i.e. publication year and ITT analysis) remained after the data on “blinding” were excluded, and their P values were 0.005 and <0.001 respectively. Table 3 depicts the results of our linear regression.

**Table 3:** Results of linear regression for predictors associated with CONSORT methodological score.

Predictor	Univariate		Multivariate	
	beta (95%CI)	P	beta (95%CI)	P
Journal	−0.027 (−0.074, 0.020)	0.257	–	–
Impact factor	−0.104 (−0.174, −0.034)	0.004	−0.054 (−0.123, 0.015)	0.232
Publication year	0.059 (0.026, 0.091)	<0.001	0.047 (0.016, 0.078)	0.004^¶^
No. of authors	0.003 (−0.036, 0.042)	0.881	–	–
Multicenter trials	−0.047 (−0.517, 0.423)	0.845	–	–
Type of interventions	−0.025 (−0.223, 0.172)	0.801	–	–
Sample size	0.000 (−0.001, 0.000)	0.155	−0.067 (−0.001, 0.000)	0.080
Funding support	0.039 (−0.047, 0.125)	0.376	–	–
Allocation concealment	−0.341 (−1.696, 1.015)	0.622	–	–
ITT analysis	0.586 (0.383, 0.788)	<0.001	0.601 (0.404, 0.799)	<0.001^¶^
Length of descriptions of interventions in words	0.000 (0.000, 0.000)	0.091	0.065 (−0.132, 0.263)	0.157
Reproducibility of interventions	0.055 (−0.153, 0.263)	0.606	–	–
Length of descriptions of controls in words	0.000 (−0.002, 0.001)	0.871	–	–
Reproducibility of control	−0.056 (−0.202, 0.091)	0.458	–	–

Note: ^¶^When data on “blinding” were deleted, two predictors associated with CONSORT score (i.e., publication year and ITT analysis) remained, and their P values were 0.005 and <0.001 respectively.

## Discussion

In this review, we investigated 680 RCT fulltexts using CONSORT methodological items and found that RCTs of nursing interventions in Chinese nursing care journals reported “trial design,” “participant settings,” changes in “outcome,” “sample size,” “allocation concealment mechanism,” “implementation,” “blinding,” and additional analyses of “statistical methods” poorly. The overall CONSORT score was low. Weak correlations between CONSORT score and two variables (i.e., publication year and ITT analysis) were found.

Concordant with our findings, Zhang et al. [11] showed that the reporting of methodological items in cancer nursing care literature was also poor in terms of outcomes, sample size, randomization sequence generation, allocation concealment mechanism, implementation, and blinding. Zhu et al. [15] demonstrated that only 3.8% of RCTs of nursing interventions described randomization sequence generation methods; furthermore, in traditional Chinese medicine nursing studies [10], 7.8% of RCTs described randomization sequence generation, 1.4% described the allocation concealment mechanism, and 3.1% described blinding. Evidence from RCTs of nursing interventions in English [13], [14], [16] also supported our findings. Samaan et al. [5] reviewed systematic reviews of adherence to reporting guidelines across different clinical areas and found a prevailing lack of reporting quality in most disciplines. Additionally, Samaan et al. [5] showed that 80% of the studies that did not adhere to CONSORT guidelines had negative conclusions, such as “some improvements are needed, reporting inadequate/poor/medium/suboptimal, etc.” Our findings are consistent with other systematic reviews in other specializations [19], [22]. Given these findings, we have provided important evidence to alert Chinese nursing researchers of the urgent need for improving the reporting of RCTs on nursing interventions. This is especially true for randomization, as we found that 87.7% (4839/5519) of RCTs that mentioned “random” in abstracts and/or fulltexts did not actually include details of the randomization or used incorrect randomized methods, such as alternate allocation and allocation by record number. Similar results were reported by Wu et al. [24].

We found poorer reporting of blinding in 83.4% of non-pharmacological interventions, but no correlation was established via the regression analysis. Due to some methodological challenges, such as the complexity of the intervention [23], expertise of the care provider [23], and difficulties with blinding [16], it is believed that non-pharmacological interventions always limit the blinding of participants and caregivers; however, the blinding of data collectors is often achievable. Regardless of whether blinding is possible, authors can and should state who was blinded. Therefore, although it seemed unnecessary in some included studies, blinding was judged as impracticable because the authors did not report its details. Our regression analysis indicated that publication year had a weak correlation with reporting quality. This demonstrates that reporting quality is improving yearly. We also examined the relationship between reporting quality and methodological quality of RCTs by regression of allocation concealment and ITT analysis. The regression indicated only weak correlations with ITT analysis, and no correlations with the other variable. In order to explore the influence of the score of “blinding (11a, 11b)” on regression analysis, data on “blinding” were deleted, and the results showed that the two same predictors (i.e. publication year and ITT analysis) were associated with CONSORT score analysis.

The main strengths of this study were the comprehensive search strategy, determination of associations of CONSORT score with various predictors, and the well-conducted quality control assessment. However, this review had some limitations. First, nursing interventions are often far more complicated than drug interventions are. We did not investigate the reporting of items related to external validity. Second, as our eligible criteria was not restricted to any specific nursing intervention such as drug interventions, social and psychological interventions, traditional Chinese medicine interventions, or complex interventions, we did not use CONSORT extensions for social and psychological interventions, non-pharmacological interventions, herbal interventions, and CReDECI criteria [25]–[28]. In addition, we did not find a pragmatic trial among the included studies, so the CONSORT extension for pragmatic trials [29] was also not used. Third, according to our eligibility criteria, no dissertation in nursing care was included. A search on the Internet in the Doctoral Dissertations and Masters' Theses sub-set of the China Knowledge Resource Integrated Database yielded 2312 citations dating up to June 2013 that mentioned “random.” Finally, because none of the studied nursing journals endorsed CONSORT as a requirement for a manuscript submission in China, we did not investigate differences in the reporting between each item by comparing CONSORT-endorsing journals with CONSORT non-endorsing journals.

## Conclusions

In conclusion, the completeness of methodological reporting of RCTs in Chinese nursing care journals was very poor, especially in terms of trial design, outcome changes, sample size calculation, allocation concealment, blinding, and statistical methods. Authors are highly recommended to use the CONSORT criteria when submitting reports of RCTs. Editors should also present their peer-reviewed manuscripts under the guidance of CONSORT to ensure that journal articles contain the key clinical and methodological details that readers need. Initiatives recommended by Altman [30] should be accepted; these suggest that authors should sign a publication transparency declaration as part of every journal submission and journals should update their instructions to authors.

## Supporting Information

Appendix S1 **The total cites and impact factors in five Chinese nursing journals.**
(DOC)

Appendix S2 **Comprehensive electronic search strategy for CBM disc.**
(DOC)

Appendix S3 **Data extraction form.**
(DOC)

Appendix S4 **Number of RCTs in each publication year.**
(TIF)

Checklist S1 **PRISMA Checklist.**
(DOC)
