# Supplementary material for: Methodological Reporting of Randomized Trials in Five Leading Chinese Nursing Journals
Source: PLoS One. 2014 Nov 21;9(11):e113002. doi: 10.1371/journal.pone.0113002 (PMC4240555; doi:10.1371/journal.pone.0113002)
Supplement: Appendix S1 — The total cites and impact factors in five Chinese nursing journals. (DOC) [file pone.0113002.s001.doc]

Appendix S1 The total cites and impact factors in five Chinese nursing journals

| Journal Name | Abbreviation | Publication years | Total cites | Impact factor* |
| --- | --- | --- | --- | --- |
| Chinese Journal of Nursing | CJN | 1954-6.2012 | 12358 | 3.458 |
| Journal of Nurses Training | JNT | 1986-6.2012 | 17257 | 1.272 |
| Chinese Journal of Practical Nursing | CJPN | 1985-6.2012 | 12225 | 0.901 |
| Chinese Journal of Modern Nursing | CJMN | 1995-6.2012 | 10772 | 0.644 |
| International Journal of Nursing | IJN | 1980-6.2012 | 6211 | 0.397 |

Note: * Although three of the five journals have an impact factor under 1.0, they are recognized as the leading nursing journals by “Chinese S&T Journal Citation Reports 2012”, which focus on both general and disease-specific topics rather than on only disease-specific topics.
