# Supplementary material for: Methodological Reporting of Randomized Trials in Five Leading Chinese Nursing Journals
Source: PLoS One. 2014 Nov 21;9(11):e113002. doi: 10.1371/journal.pone.0113002 (PMC4240555; doi:10.1371/journal.pone.0113002)
Supplement: Appendix S2 — Comprehensive electronic search strategy for CBM disc. (DOC) [file pone.0113002.s002.doc]

Appendix S2 Comprehensive electronic search strategy for CBM disc*

#1 random$

#2 randomized controlled trial/

#3 randomized controlled trial$.pt.

#4 double blind

#5 double blind method/

#6 single blind

#7 single blind method/

#8 triple blind

#9 blind$

#10 #1 OR #2 OR #3 OR #4 OR #5 OR #6 OR #7 OR #8 OR #9

#11 #10/limit:animal

#12 #10 NOT #11

#13 ‘Chinese Journal of Nursing’ [journal searching]

#14 ‘Chinese Journal of Modern Nursing’ [journal searching]

#15 ‘International Journal of Nursing’ [journal searching]

#16 ‘Chinese Journal of Practical Nursing’ [journal searching]

#17 ‘Journal of Nurses Training’ [journal searching]

#18 #13 OR #14 OR #15 OR #16 OR #17

#19 #12 AND #18

Note: * CBM disc is offered by Chinese Biomedicine Literature Database covering literature published in Chinese biomedicine journals from 1978 to present and is used commonly in China like Pubmed. All terms mentioned above are in Chinese.
