# Supplementary material for: Methodological Reporting of Randomized Trials in Five Leading Chinese Nursing Journals
Source: PLoS One. 2014 Nov 21;9(11):e113002. doi: 10.1371/journal.pone.0113002 (PMC4240555; doi:10.1371/journal.pone.0113002)
Supplement: Appendix S3 — Data extraction form. (DOC) [file pone.0113002.s003.doc]

**Appendix S3 Data Extraction Form (English version)**

| **ID** | | **Answers** | | |
| --- | --- | --- | --- | --- |
| Date | | __/__/__ (M/D/Y) | | |
| Author/s | | _________________ | | |
| Journal information | |  | | |
| Journal name | |  | | |
| Impact factor (IF) | |  | | |
| **General characteristics** | | **Descriptions** | | |
| Eligible study title | |  | | |
| First author | | Name _________ | | |
| Publication year | | Year _________ | | |
| Study design | |  | | |
| Number of authors | | Number _________ | | |
| Multicenter trials | |  | | |
| Types of interventions | | 🞎 Non pharmacologic interventions  🞎 Pharmacologic interventions | | |
| Sample size | |  | | |
| Funding source | |  | | |
| Length of descriptions of interventions and controls in words | | Interventions _________ words  Controls _________ words | | |
| Reproducibility of the interventions and controls^*^ | | Interventions: Yes 🞎 No 🞎  Controls: Yes 🞎 No 🞎 | | |
| Allocation concealment | | Yes 🞎 No 🞎 | | |
| Intent-to-treatment (ITT) analysis | | Yes 🞎 No 🞎 | | |
| Randomization methods | | Valid 🞎 Invalid 🞎  Please specify: | | |
| Valid RCT | | Yes 🞎 No 🞎 | | |
| **If a trial provides the randomization methods, please finish the following form.** | | | | |
| **Items** | **CONSORT** | | | |
|  | **Explanation** of items** | | **Y/N** | **Please specify** |
| Trial design (item 3a) | Description of trial design (such as parallel, factorial) including allocation ratio | |  |  |
| Trial design (item 3b) | Important changes to methods after trial commencement (such as eligibility criteria), with reasons | |  |  |
| Participants (item 4a) | Eligibility criteria for participants | |  |  |
| Participants (item 4b) | Settings and locations where the data were collected | |  |  |
| Interventions (item 5) | The interventions for each group with sufficient details to allow replication, including how and when they were actually administered | |  |  |
| Outcomes (item 6a) | Completely defined pre-specified primary and secondary outcome measures, including how and when they were assessed | |  |  |
| Outcomes (item 6b) | Any changes to trial outcomes after the trial commenced, with reasons | |  |  |
| Sample size (item 7a) | How sample size was determined | |  |  |
| Sample size (item 7b) | When applicable, explanation of any interim analyses and stopping guidelines | |  |  |
| Randomization sequence  generation (item 8a) | Method used to generate the random allocation sequence | |  |  |
| Randomization sequence  generation (item 8b) | Type of randomisation; details of any restriction (such as blocking and block size) | |  |  |
| Allocation concealment  mechanism (item 9) | Mechanism used to implement the random allocation sequence (such as sequentially numbered containers), describing any steps taken to conceal the sequence until interventions were assigned | |  |  |
| Implementation (item 10) | Who generated the random allocation sequence, who enrolled participants, and who assigned participants to interventions | |  |  |
| Blinding (item 11a) | If done, who was blinded after assignment to interventions (for example, participants, care providers, those assessing outcomes) and how | |  |  |
| Blinding (item 11b) | If relevant, description of the similarity of interventions | |  |  |
| Statistical methods  (item 12a) | Statistical methods used to compare groups for primary and secondary outcomes | |  |  |
| Statistical methods  (item 12b) | Methods for additional analyses, such as subgroup analyses and adjusted analyses | |  |  |
| In total (score) |  | | | |

Note: * Reproducibility of the interventions and controls were subjectively assessed by authors based on whether the interventions and controls were described in enough details to be reproducible;

**explanation is given according to Schulz KF, et al. (2010) [6].
