# Supplementary figures and images for: Methodological Reporting of Randomized Trials in Five Leading Chinese Nursing Journals
Source: PLoS One. 2014 Nov 21;9(11):e113002. doi: 10.1371/journal.pone.0113002 (PMC4240555; doi:10.1371/journal.pone.0113002)

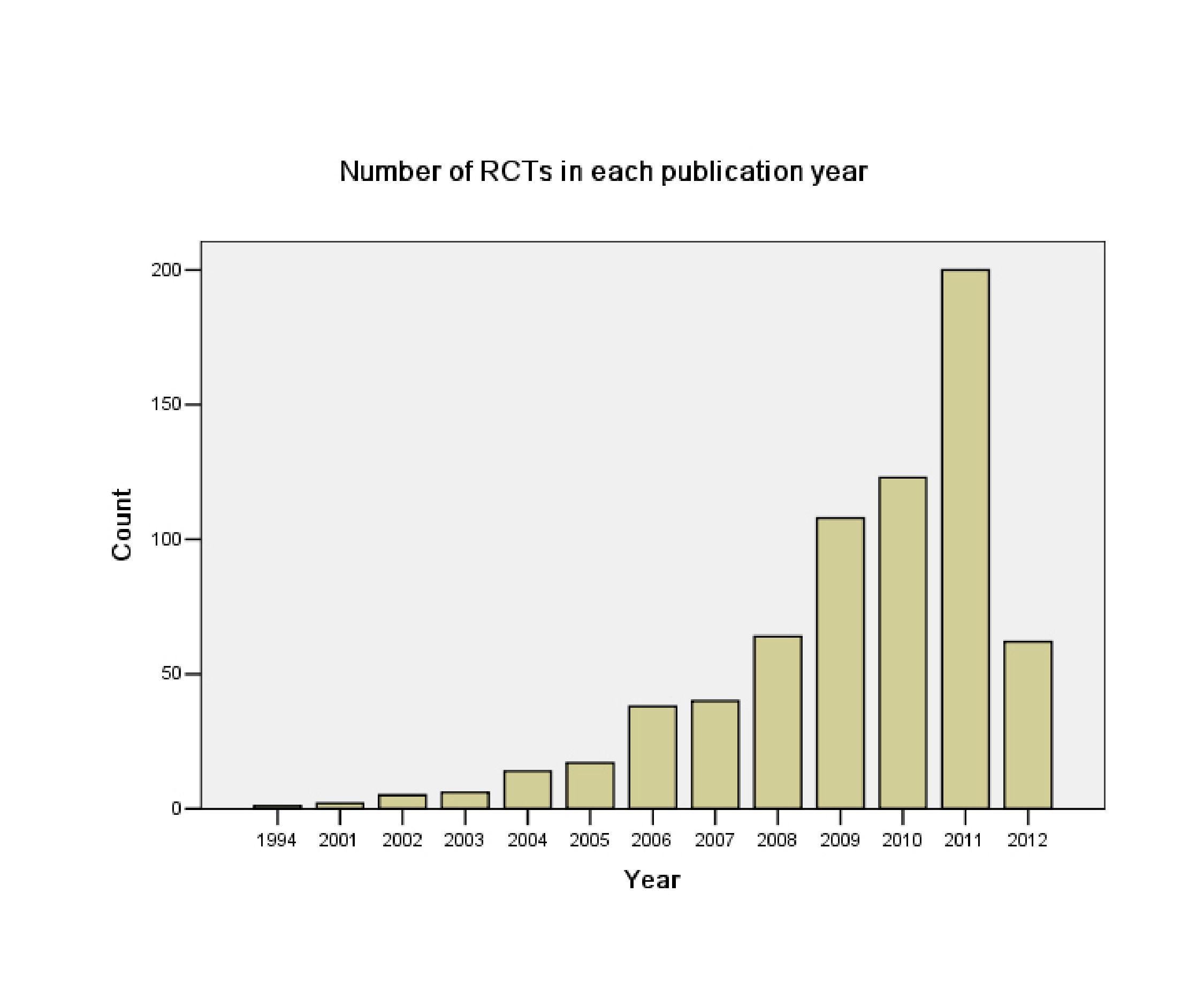

Supplement: Appendix S4 — Number of RCTs in each publication year. (TIF) [file pone.0113002.s004.tif]
